# Anxiety Specific Response and Contribution of Active Hippocampal Neural Stem Cells to Chronic Pain Through Wnt/β-Catenin Signaling in Mice

**DOI:** 10.3389/fnmol.2018.00296

**Published:** 2018-08-24

**Authors:** Youyi Zhao, Li Zhang, Mengmeng Wang, Jianping Yu, Jiping Yang, Aidong Liu, Han Yao, Xinyu Liu, Yahui Shen, Baolin Guo, Yazhou Wang, Shengxi Wu

**Affiliations:** ^1^Department of Neurobiology and Institute of Neurosciences, School of Basic Medicine, Fourth Military Medical University, Xi’an, China; ^2^School of Basic Medicine, Chengdu Medical College, Chengdu, China; ^3^Department of Anatomy, Institute of Basic Medical Science, Xi’an Medical University, Xi’an, China; ^4^Department of Neurobiology, The First Affiliated Hospital of Chengdu Medical College, Chengdu, China

**Keywords:** chronic pain, anxiety, Wnt/β-catenin signaling, hippocampus, adult neural stem cell

## Abstract

Chronic pain usually results in persistent anxiety, which worsens the life quality of patients and complicates the treatment of pain. Hippocampus is one of the few brain regions in many mammalians species which harbors adult neural stem cells (NSCs), and plays a key role in the development and maintenance of chronic anxiety. Recent studies have suggested a potential involvement of hippocampal neurogenesis in modulating chronic pain. Whether and how hippocampal NSCs are involved in the pain-associated anxiety remains unclear. Here, we report that mice suffering persistent neuropathic pain showed a quick reduction of active NSCs in the ventral dentate gyrus (vDG), which was followed by the decrease of neurogenesis and appearance of anxiety. Wnt/β-catenin signaling, a key pathway in sustaining the active status of NSCs was suppressed in the vDG of mice suffering chronic pain. Depleting β-catenin by inducible Nestin-Cre significantly reduced the number of active NSCs and facilitated anxiety development, while expressing stabilized β-catenin amplified active NSCs and alleviated anxiety, indicating that Wnt activated NSCs is required for anxiety development under chronic pain. Treatment with Fluoxetine, the most widely used anxiolytic in clinic, significantly increased the proliferation of active NSCs and enhanced Wnt signaling. Interestingly, both β-catenin manipulation and Fluoxetine treatment had no significant effects on the pain thresholds. Therefore, our data demonstrated an anxiety-specific response and contribution of activated NSCs to chronic pain through Wnt/β-catenin signaling, which may be targeted for treating chronic pain- or other diseases-associated anxiety.

## Introduction

Over half of the patients who suffer from chronic pain develop mood disorders such as anxiety and depression (McWilliams et al., 2003; Maletic and Raison, 2009; Yalcin et al., 2014), which make the treatment of chronic pain more complicated and increase the expenses of medication. However, for a long time, investigations on pain and anxiety have been mostly carried out independently. Recent human imaging studies have revealed pain-anxiety-interactions involving brain regions such as amygdala, anterior cingulate cortex (ACC) and hippocampus (Liberzon and Martis, 2006; Zhuo, 2016). Many studies have demonstrated that amygdala may be a core region for acute anxiety (Neugebauer, 2015), while ACC and hippocampus for the chronic anxiety (Zhuo, 2017). At the molecular level, changes of synaptic plasticity, disturbance of serotoninergic or GABAergic transmitters and dysregulation of hypothalamus-pituitary-adrenocortical (HPA)-hormones have been suggested to account for the development of anxiety (Faravelli et al., 2012; Bliss et al., 2016; Craske and Stein, 2016; Pizzo et al., 2018). However, all the clinic-in-use anxiolytics targeting the above mechanisms require at least 3–4 weeks’ drug administration to alleviate anxiety, suggesting that other mechanisms may also be involved in the development of anxiety.

Existence of slow neurogenesis is a unique feature of hippocampus in many mammalian species, which offers a new dimension of neural plasticity to pathological stimulations. The time course of hippocampal neurogenesis and its response to stress fit well with the slow effectiveness of anti-anxietics. A “neurogenic” hypothesis has recently been proposed as a potential mechanism for the disease-associated anxiety (Miller and Hen, 2015). In case of chronic pain, previous studies have indicated that hippocampal neurogenesis may be involved in the modulation of pain perception (Apkarian et al., 2016; Zheng et al., 2017). It is thus possible that hippocampal neural stem cells (NSCs) may play a role in the chronic pain-associated anxiety, which however, remains largely unclear.

There are two populations of NSCs in hippocampus, namely the quiescent and active NSCs (Wang et al., 2011). The quiescent NSCs have longer cell cycle time while the active NSCs proliferate more actively and respond to stimulus more quickly (Rolando and Taylor, 2014; Goncalves et al., 2016). The quiescent NSCs exhibit radial morphology with long processes extending into the granular zone, and the active NSCs mostly show horizontal morphology located at the basal of granular zone (Lugert et al., 2010). Both populations of NSCs are maintained by distinct niche signals (Wang et al., 2011). In the present study, we explored whether the active NSCs played a role in the chronic pain-associated anxiety since they respond to pathological stimulations more actively, by focusing on Wnt/β-catenin signaling, a key signaling pathway in regulating the proliferation and neuronal differentiation of active NSCs (Wang et al., 2011; Goncalves et al., 2016). Our data revealed an anxiety specific response and contribution of active NSCs to chronic pain, possibly through Wnt/β-catenin signaling.

## Materials and Methods

### Mice and Reagents

This study was carried out in accordance with the recommendations of the guidelines for experimental animal care and use by the Committee of the Animal Care and Use Committee of Fourth Military Medical University. The protocol of animal experiments was approved by the Committee of the Animal Care and Use Committee of Fourth Military Medical University.

Male C57BL/6 mice (8–12 weeks old, 25–30 g) were used for pain-modeling or as wild type control. Top gal mice (DasGupta and Fuchs, 1999), β-cateninEX3^loxp/+^ (Harada et al., 1999), and β-catenin^loxp/loxp^ (Brault et al., 2001) mice were bought from Jackson lab. Generation of Nestin-CreER mice was described previously (Kageyama et al., 2008). Tamoxifen, BrdU and Fluoxetine were bought from Sigma.

### Spared Nerve Injury and Mice Treatment

Spared nerve injury (SNI) and sham surgery were made as described previously with minor modifications (Decosterd and Woolf, 2000). Briefly, mice were anesthetized by 1% Pentobarbital plus Ether inhalation. Before surgery, tail response to a stab was tested. Skin on the lateral surface of the right thigh was incised. The sciatic nerve and its three branches were exposed by a blunt dissection through the biceps femoris muscle. Distal to the trifurcation of its branches, the common peroneal and the tibial nerves were ligated by using 7.0 silk and axotomized, removing a 2–4 mm piece of each distal nerve stump. Care was taken to keep the sural nerve untouched. After nerve injury, incisions were closed with muscle and skin sutures. Surgery was finished within 15 min. In sham surgery, the sciatic nerve branches were exposed, but not injured.

Acute BrdU incorporation: three shots of BrdU were injected with an interval of 2 h. Two hours after the last injection, mice were sacrificed.

Chronic BrdU labeling: BrdU was injected for 14 successive days from the day of SNI, and mice were sacrificed at 21 dpi to assess the neurogenesis in wild-type (WT), conditioned β-catenin knockout or overexpression mice. BrdU was injected for seven successive days from 14 dpi and mice were sacrificed at 21 dpi to assess the effects of Fluoxetine on neurogenesis.

### Immunohistochemistry

Animals were sacrificed and perfused intracardially with 4% cold paraformaldehyde phosphate buffer (pH 7.4). Brain tissue was cryoprotected by 25% sucrose. For each mouse, serial sections (20 μm in thickness for each section) were cut and all the sections were collected onto eight slides. For immunostaining, the sections were blocked by 0.01 M phosphate buffered saline (PBS) containing 0.3% Triton X-100 and 3% bovine serum albumin (BSA) for 1 h. Primary antibodies were used as following: guinea pig anti-DCX (1:500, Millipore), rat anti-BrdU (1:200, Abcam), rabbit anti-NeuN (1:500, TEMECULA), goat anti-Nestin (1:500, Santa Cruz), rabbit anti-β-gal (1:500, MP), anti-β-catenin (1:200, Millipore). After primary antibodies incubation and washing with PBS, sections were incubated with their corresponding secondary antibodies conjugated with Alexa Fluor 594 or Alexa Fluor 488 (Jackson Immunoresearch) for 2–4 h at room temperature protected from light. The nuclei were counterstained by Hoechst33342 (1:5,000, Sigma). All immunostained sections were photographed under a confocal microscope (FV1000, Olympus) with same setting.

For the immunostaining of BrdU, sections were treated with 2 N HCl for 30 min at 37°C, and then washed with borate buffer (pH.8.5) before incubation with primary antibody.

Horizontal and radial NSCs were identified by the morphology of Nestin-positive cells as described (Lugert et al., 2010; Mira et al., 2010). Briefly, the bipolar shaped Nestin-positive cells which localize at and line in parallel along the basal granular layer of dentate gyrus were identified as horizontal NSCs. The cells which show a long process vertical to the basal granular layer were identified as radial NSCs.

For the quantification of NSCs and new neurons, immune-positive cells were quantified in dentate gyrus per mm as described (Antonelli et al., 2018).

### Behavior Assays

All behavioral tests were performed by an experienced investigator blinded to mice treatment and genotype.

#### Mechanical Allodynia

Mechanical threshold was evaluated before SNI, 1 day, 3 days, 5 days, 7 days, 10 days, 14 days and 21 days post SNI. Mice were habituated to the behavioral testing paradigms for 3–5 days before initiating data collection. Mice were placed on an elevated wire grid and the plantar surface of the paw was stimulated with a series of ascending force von Frey monofilaments) 0.008 g, 0.02 g, 0.04 g, 0.07 g, 0.16 g, 0.4 g, 0.6 g and 1 g). The threshold was taken as the lowest force that evoked a brisk withdrawal response to one of five repetitive stimuli (Tal and Bennett, 1994). The lateral, medial plantar and dorsal surface of the paw was tested. The withdrawal threshold was measured five times and expressed as the tolerance level in grams.

#### Thermal Hyperalgesia

Thermal threshold was evaluated before SNI, 1 day, 3 days, 5 days, 7 days, 10 days, 14 days and 21 days post SNI. Mice were placed in a transparent Perspex box on a thin glass platform, and habituated for 1 h before initiating data collection. An infrared heat lamp device was then positioned underneath the targeted hind paw to direct a focused radiant light source from below the glass onto the plantar surface of one hindpaw. The paw withdrawal latency was determined with a 5-min inter-trial interval. The infrared intensity was set at 25, which produced baseline paw withdrawal latencies of 20–25 s. The mean latency of withdrawal was determined by five tests. A cut-off of 30 s was used.

#### Open Field Test

The open field test was carried out in a white opaque plastic chamber (50 × 50 × 35 cm) at 10 or 21 dpi as described in the previous study (Huang et al., 2015) with slight modification. The open field was divided into 25 squares with same area. The central nine squares in were defined as central area, and the remaining as periphery area. For each test, mouse was gently placed in one corner, and the movement was recorded for 5 min with a video tracking system. The time spent and distance traveled in the central area and the total distance traveled in the field were measured using the SMART software (SMART 3.0, Panlab S.L.U.). Between each test, 75% ethanol was used to clean the open field area.

#### Elevated Plus Maze Test

The elevated plus maze test was performed on the next day of the open-field test. The maze was placed 50 cm above the floor and consisted of two open arms and two closed arms (30 × 5 cm and 15 cm wall height for the closed arms). Each mouse was placed onto the center area, heading toward the same open arm, and videotaped in the following 5 min. The time spent and moving distance in the open arms, and the total movements in both open and closed arms were analyzed using the software SMART 3.0. The maze was cleaned by 75% ethanol between tests.

### Western-Blotting

Hippocampal tissues were isolated and lysed by RIPA buffer at the presence of proteinase inhibitors. Protein concentration was determined by BCA assay. Then proteins were separated in 10% acrylamide gels by SDS-PAGE and transferred to PVDF membrane. Membranes were blocked in TBS containing 0.1% (v/v) Tween 20 and 5% (w/v) nonfat skim milk before incubation with primary antibodies. The following antibodies were used: rabbit anti-β-gal (1:1,000, CST), rabbit anti-β-catenin (1:600, Millipore), rabbit anti-Axin2 (1:1,000, Abcam), mouse anti-Actin (1:5,000, Sigma) After three washes with TBS-T, membranes were incubated with HRP-conjugated anti-mouse or anti-rabbit secondary antibodies (1:5,000; Zhuangzhi Biotech Co., Ltd.). Bands were visualized by ECL.

### Data Analysis

For immunohistochemistry and Western blots, at least three biological repeats were performed for each experiment. For behavior study, at least five mice were included per group. The data were presented as means ± standard error (SE). For the pain thresholds, data were analyzed by one-way analysis of variance (ANOVA), followed by Dunnett *post hoc* using SPSS l 6.0 (Chicago, IL, USA). For all the other experiments, the data were analyzed by unpaired, two-tailed Student’s *t*-test. *P* values less than 0.05 were considered as statistical significant.

## Results

### Chronic Neuropathic Pain Reduces Active NSCs in Ventral Dentate Gyrus (vDG) and Induces Anxiety

**Figure 1:**
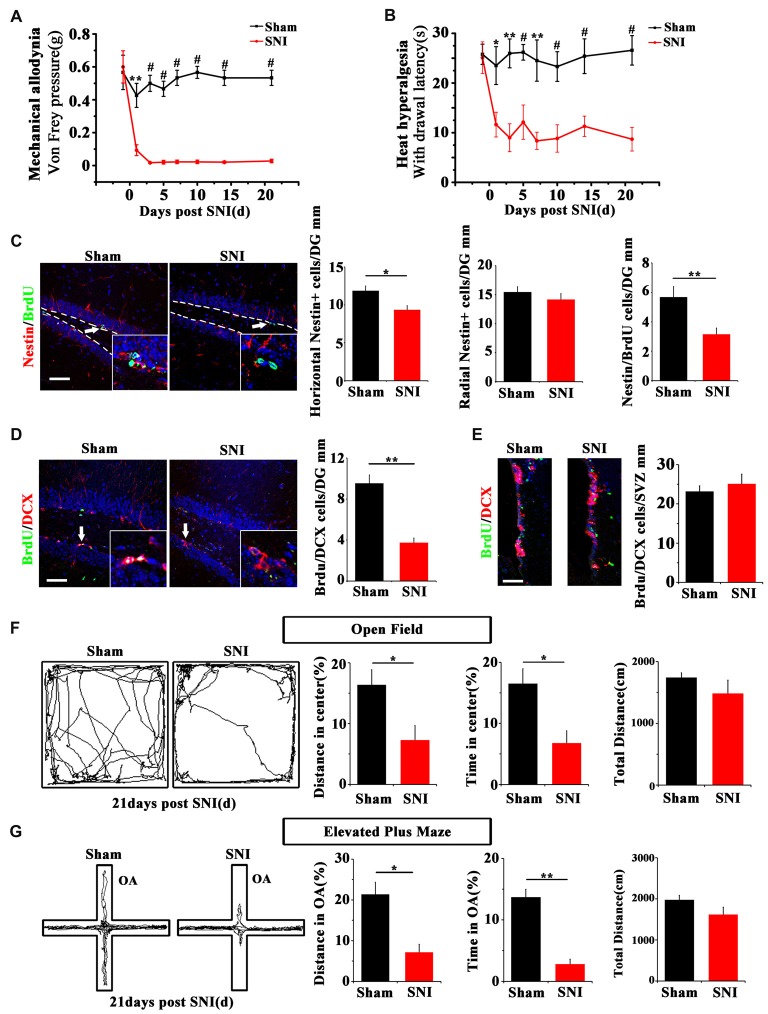
Effects of chronic pain on the activation of neural stem cells (NSCs) in ventral hippocampus and the appearance of anxiety. **(A)** Pain threshold for mechanical allodynia after spared nerve injury (SNI). **(B)** Pain threshold for thermal hyperalgesia after SNI. Notice the persistence of chronic pain to at least 21 days after SNI. **(C)** Double-immunostaining of Nestin/BrdU and the quantification at 7 dpi after acute BrdU incorporation. Notice the decrease of horizontal Nestin-positive cells, which were the major BrdU-labeled cells in SNI-treated mice. **(D)** Double-immunostaining and quantification of DCX/BrdU-positive cells in ventral dentate gyrus (vDG) at 21 dpi. **(E)** Double-immunostaining and quantification of DCX/BrdU-positive cells in subventricular zone (SVZ) at 21 dpi. Notice the decrease of DCX/BrdU-positive cells in vDG and no change of DCX/BrdU-positive cells in SVZ between sham and SNI-treated mice. **(F)** Open field assay of sham-injured and SNI mice. **(G)** Elevated plus maze test of sham-injured and SNI mice. Notice the decrease of movements of SNI-treated mice in the open field and open arm. Bars = 50 μm in **(C–E)**. OA, open arm. Inserts in **(C,D)** are magnified typical double-stained cells, which were pointed by arrows. Dashed lines in **(C)** showed the basal line along which quantification made. Values represent mean ± SE. One-way analysis of variance (ANOVA) analysis with Dunnett’s *post hoc* test was performed in **(A,B)**. Unpaired, two tailed Student’s *t*-tests were performed in **(C–G)**. *P < 0.05, **P < 0.01, ^#^*P* < 0.001.

SNI was adopted to produce chronic pain. Persistent mechanical allodynia and thermal hyperalgesia were detected for over 3 weeks (*P* < 0.05, *n* = 5 mice per group; Figures 1A,B). Because the ventral hippocampus is thought as a core region in mood regulation (Fanselow and Dong, 2010), we focused mainly on the ventral hippocampus in the present study. The response of hippocampal NSCs to chronic pain was first examined. The results showed a significant reduction of horizontal Nestin+ active NSCs in the ventral dentate gyrus (vDG) at 7 dpi (*P* = 0.006, *n* = 5 mice per group), while the radial Nestin+ quiescent NSCs remain unchanged (Figure 1C). To further confirm the changes of active NSCs, we performed acute BrdU-incorporation assay. The results showed that most of the BrdU-positive cells after acute administration were horizontal Nestin+ cells. The number of BrdU/Nestin-positive cells decreased from 5.7 ± 0.7 cells/mm in the vDG of sham-injured mice to 3.2 ± 0.4 cells/mm in the vDG of SNI-treated mice (*P* = 0.012, *n* = 5 mice per group; Figure 1C). To evaluate the changes of neurogenesis, we performed chronic BrdU-labeling. At 21 dpi, the number of DCX/BrdU-positive cells decreased significantly in the vDG, as compared with that in sham-injured mice (*P* = 0.0011, *n* = 5 mice per group; Figure 1D). Interestingly, no change of BrdU/DCX-positive cells in the subventricular zone (SVZ) was found in SNI-treated mice at 21 dpi, as compared with sham control (Figure 1E). These data indicated that chronic pain reduced the number of hippocampal active NSCs.

We next investigated whether chronic pain could induce anxiety in mice. At 21 dpi, in comparison with sham injured mice, SNI-treated mice showed significant reduction of moving distance (*P* = 0.014, *n* = 5) and time spent (*P* = 0.001, *n* = 5 mice per group) in the center of open field (Figure 1F). In elevated plus maze test, the moving distance and time spent in the open arm of SNI-treated mice were significantly decreased (*P* = 0.029 and *P* = 0.017 respectively, *n* = 5 mice per group), as compared with sham-injured control (Figure 1G). No significant difference of the total movement, time resting/moving and average moving speed were found in both open field and elevated plus maze tests between sham-injured and SNI-treated mice (Figures 1F,G and Supplementary Figure S1). These data demonstrated that the anxiety-like behavior appears after a drop of active hippocampal NSCs in mice suffering chronic pain.

To directly investigate the link between NSCs reduction and the development of pain-associated anxiety, we used Nestin-CreER:ROSA-DTA mice to ablate Nestin-positive cells by treating the mice with Tamoxifen (TAM) for five successive days before pain-induction (Supplementary Figure S2A). To assess the recombinant efficiency, Nestin-CreER:ROSA-YFP mice were treated with same TAM injection protocol. The results showed that approximately 84% Nestin-positive cells were labeled by YFP (Supplementary Figure S2B). In Nestin-CreER:ROSA-DTA mice, the NSCs and behavior were analyzed at 10 dpi when most of the SNI-treated WT mice do not show anxiety. The results showed that TAM led to a significant decrease of DCX-positive cells and an early onset of anxiety at 10 dpi as compared with those TAM-treated WT mice (Supplementary Figures S2C,D). These data indicated a role of NSCs and subsequent neurogenesis in the pain-associated anxiety.

### Chronic Neuropathic Pain Suppresses Wnt/β-Catenin Signaling in Active NSCs in Ventral Hippocampus

**Figure 2:**
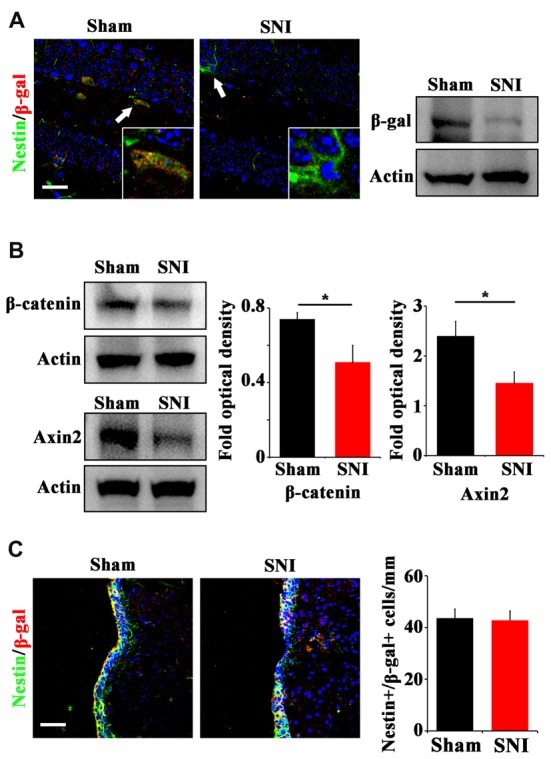
Effects of chronic pain on Wnt activity in ventral hippocampus. **(A)** Double-immunostaining of Nestin/β-gal and Western-blotting of β-gal in the ventral hippocampus of sham-injured and SNI-treated Topgal mice at 21 dpi. **(B)** Western-blotting of β-catenin and Axin2 in the ventral hippocampus of sham-injured and SNI-treated mice at 21 dpi. **(C)** Double-immunostaining and quantification of Nestin/β-gal in the SVZ of sham-injured and SNI-treated Topgal mice at 21 dpi. Inserts in **(A)** are magnified typical Nestin/β-gal-positive cells in each group, which were pointed by arrows. Bars = 25 μm in **(A)** and 50 μm in **(C)**. Values represent mean ± SE. Unpaired, two tailed Student’s *t*-tests were performed in **(B,C)**. *P < 0.05.

To explore the possible mechanism underlying the reduction of active NSCs, we focused on Wnt/β-catenin signaling, which has been demonstrated to play a key role in sustaining the active status of NSCs in adult hippocampus (Pozniak and Pleasure, 2006; Wang et al., 2011). We adopted a canonical Wnt signaling reporter mouse line, Topgal mice (DasGupta and Fuchs, 1999) and examined the expression of Wnt reporting gene β-gal after SNI. Immunohistochemistry showed that in normal mice, β-gal is mainly expressed in horizontal Nestin-positive progenitors in DG, indicating that Wnt/β-catenin signaling is activated in active hippocampal NSCs (Figure 2A). In SNI-treated mice, both immunohistochemistry and Western-blotting showed that the expression of β-gal in hippocampus was lowered remarkably at 21 dpi, as compared with that in sham-injured control (Figure 2A). Furthermore, western-blotting demonstrated a decrease of β-catenin (a key intracellular molecule of Wnt signaling) by approximately 33% (*P* = 0.044, *n* = 4 mice per group) and a decrease of Axin2 (a Wnt signaling target gene) by approximately 44% (*P* = 0.029, *n* = 4 mice per group) in the hippocampus of SNI-treated mice (Figure 2B). To confirm the downregulation of Wnt signaling, we examined the expression of Axin2 mRNA. Real-time RT-PCR revealed a significant lower level of Axin2 in the hippocampus of SNI-treated mice (Supplementary Figure S3). Interestingly, no changes of β-gal expression were found in the SVZ of SNI-treated mice, as compared with that in sham-injured mice (Figure 2C). These data indicated that chronic pain inhibits Wnt/β-catenin signaling in the active hippocampal NSCs.

### Expression of Stabilized β-Catenin in Nestin-Positive Cells Alleviates Anxiety and Amplifies Active NSCs in SNI-Treated Mice

**Figure 3:**
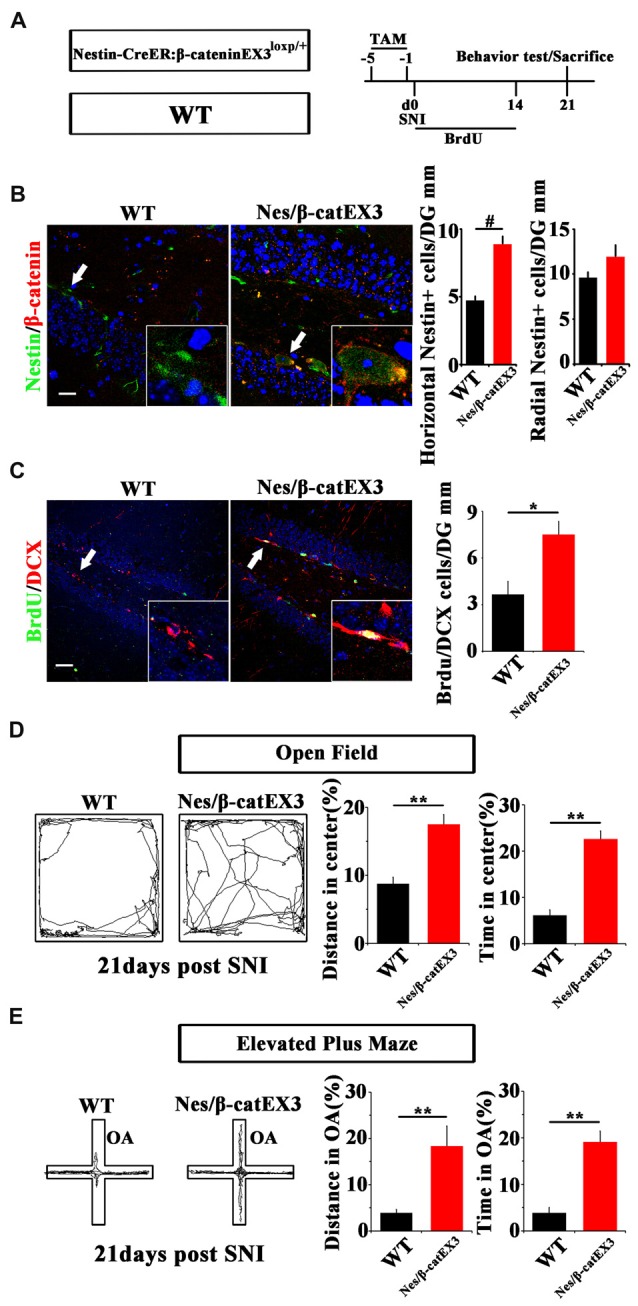
Effects of expressing stabilized β-catenin in adult neural progenitors on hippocampal neurogenesis and pain-associated anxiety. **(A)** Schematic drawing of the experimental design. **(B)** Double-immunostaining of Nestin/β-catenin and quantification of Nestin-positive cells SNI-treated wild-type (WT) or Nestin-β-catEX3 mice. Notice the enhanced expression of β-catenin and the increase of horizontal Nestin-positive cells in the hippocampus of Nestin-β-catEX3 mice. **(C)** Double-immunostaining and quantification of BrdU/DCX in SNI-treated WT or Nestin-β-catEX3 mice. **(D,E)** Open field and elevated plus maze tests of SNI-treated WT or Nestin-β-catEX3 mice at 21 dpi. Notice the alleviation of anxiety in Nestin-β-catEX3 mice with chronic pain. OA, open arm. Inserts in **(B,C)** are magnified typical double-stained cells in each group, which were pointed by arrows. Bars = 25 μm in **(B)** and 50 μm in **(C)**. Values represent mean ± SE. Unpaired, two tailed Student’s *t*-tests were performed in **(B,C)**. *P < 0.05, **P < 0.01, ^#^*P* < 0.001.

Since Wnt/β-catenin signaling was suppressed under chronic pain, we next examined the effects of over-activating Wnt/β-catenin signaling in neural progenitor cells on the pain-induced anxiety. A mouse line in which the exon-3 of β-catenin was flanked by loxp sites (β-cateninEX3^loxp/+^; Harada et al., 1999) was crossed with Nestin-CreER mice. In the resulting Nestin-CreER:β-cateninEX3^loxp/+^ (Nestin/β-catEX3) mice, TAM treatment will deplete the exon3 of β-catenin in Nestin-positive cells. Because the phosphorylation sites in the exon3 of β-catenin are essential for the degradation of β-catenin, the lack of exon3 leads to the stabilization of β-catenin in cells, thereby over-activating Wnt/β-catenin signaling in Nestin-positive cells. TAM was administered for five successive days before SNI. Animal behavior and NSCs proliferation were analyzed at 21 dpi (Figure 3A). Immunohistochemistry confirmed the increase of β-catenin in Nestin-positive cells (Figure 3B). Further analysis showed that the number of horizontal Nestin-positive cells increased significantly while the number of radial Nestin-positive cells remained unchanged in the vDG of Nes/β-catEX3 mice (*P* = 0.0003, *n* = 4 mice per group; Figure 3C). These results indicated that over-expressing β-catenin in Nestin-positive cells mainly amplified active NSCs. Accordingly, the number of BrdU/DCX-positive cells increased significantly in Nes/β-catEX3 mice as compared to TAM treated WT control (*P* = 0.023, *n* = 4 mice per group; Figure 3C).

Elevated plus maze and open field tests showed that in comparison with WT SNI-treated mice, Nes/β-catEX3 mice subjected to SNI showed significantly more movements both in the center of open field (*P* = 0.002 for moving distance, *P* = 0.001 for time spent, *n* = 5 mice per group) and the open arm (*P* = 0.019 for moving distance, *P* = 0.002 for time spent, *n* = 5 mice per group) without significant change of total movements (Figures 3D,E). No difference of basal anxiety behavior was found between TAM treated naïve WT and Nes/β-catEX3 mice, although neurogenesis was enhanced in naïve Nes/β-catEX3 mice (Supplementary Figure S4). These data indicated that enhancing Wnt/β-catenin signaling in neural progenitors could attenuate the development of anxiety under chronic pain.

### Depletion of β-Catenin in Nestin-Positive Cells Facilitates Anxiety Development and Reduces Active NSCs

**Figure 4:**
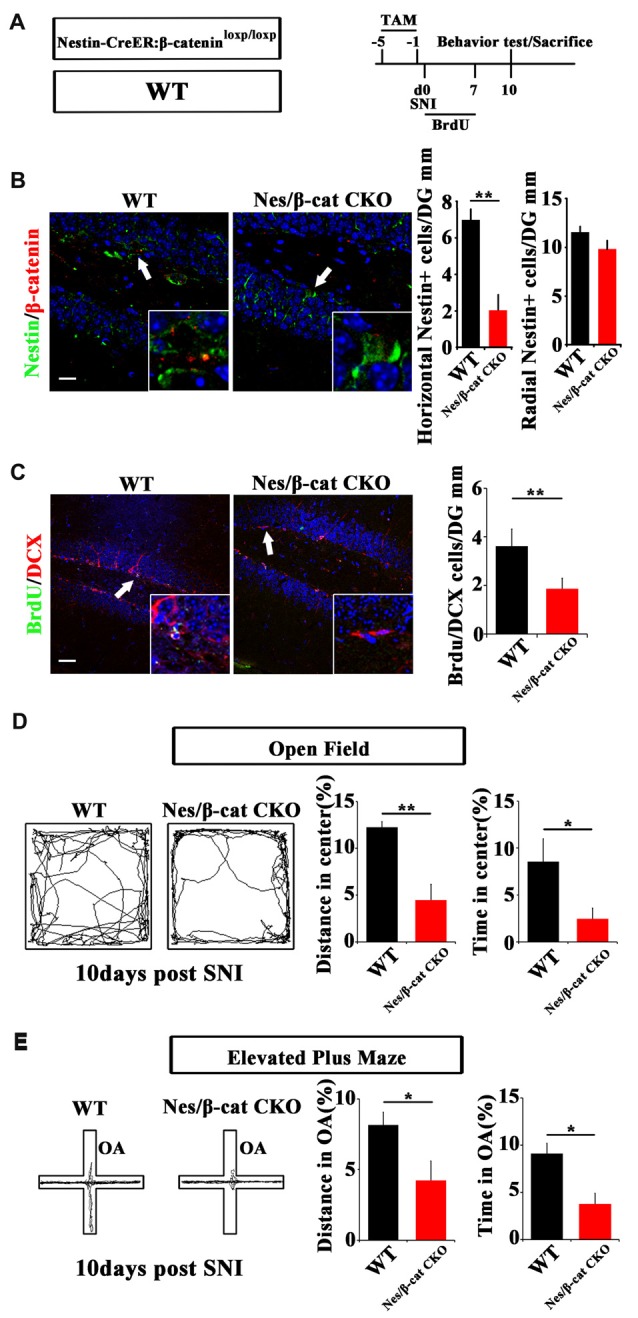
Effects of ablating β-catenin in adult neural progenitors on hippocampal neurogenesis and pain-associated anxiety.** (A)** Schematic drawing of the experimental design. **(B)** Double-immunostaining of Nestin/β-catenin and quantification of Nestin-positive cells in SNI-treated WT or Nestin-β-cat CKO mice at 10 dpi. Notice the decrease of β-catenin immunoreactivity and radial Nestin-positive cells in the hippocampus of Nestin-β-cat CKO mice. **(C)** Double-immunostaining and quantification of BrdU/DCX in SNI-treated WT or Nestin-β-cat CKO mice at 10 dpi. **(D,E)** Open field and elevated plus maze tests of SNI-treated WT or Nestin-β-cat CKO mice at 10 dpi. Notice the appearance of anxiety in Nestin-β-cat CKO mice at 10 dpi. OA, open arm. Inserts in **(B,C)** are magnified typical double-stained cells in each group, which were pointed by arrows. Bars = 25 μm in **(B)** and 50 μm in **(C)**. Values represent mean ± SE. Unpaired, two tailed Student’s *t*-tests were performed in **(B–E)**. *P < 0.05, **P < 0.01.

We next examined the effects of depleting β-catenin on pain-associated anxiety. Nestin-CreER:β-catenin^loxp/loxp^ mice (Nes/β-cat CKO) were used to ablate β-catenin in adult neural progenitors. TAM was administered for five successive days before SNI. Behavior and neurogenesis were analyzed at 10 dpi (Figure 4A). Depletion of β-catenin in Nestin-positive cells was confirmed by immunohistochemistry (Figure 4B). In contrast to the results obtained from Nes/β-catenin EX3 mice, the horizontal Nestin-positive cells and the number of DCX/BrdU-positive cells decreased more severely in Nes/β-catenin CKO mice than that of WT control (*P* = 0.0014, *P* = 0.003 respectively, *n* = 4 mice per group; Figures 4B,C).

Open field test showed that, at 10 days after SNI-treatment when most of the WT mice do not show anxiety behavior, Nes/β-cat CKO mice showed approximately 64% reduction of moving distance (*P* = 0.005, *n* = 5) and approximately 72% reduction of time spent (*P* = 0.027, *n* = 5) in the center of open field without significant change of total movement (data not shown), as compared with WT control (Figure 4D). Elevated plus maze test showed that the moving distance and time spent of Nes/β-cat CKO mice in the open arm were decreased by approximately 48% and 59% respectively without significant change of total movement (*P* = 0.042, *P* = 0.011 respectively. *n* = 5 mice per group; Figure 4E). In regard of basal anxiety behaviors, no significant difference was detected between Nes/β-cat CKO and WT mice (Supplementary Figure S5), which was consistent with previous report (Petrik et al., 2012; Yun et al., 2016). These data indicated that depleting β-catenin in adult neural progenitors could impair the proliferation of active hippocampal NSCs and facilitate development of anxiety under chronic pain.

### Fluoxetine Treatment Amplifies Active NSCs and Enhances Wnt Signaling in Hippocampus

**Figure 5:**
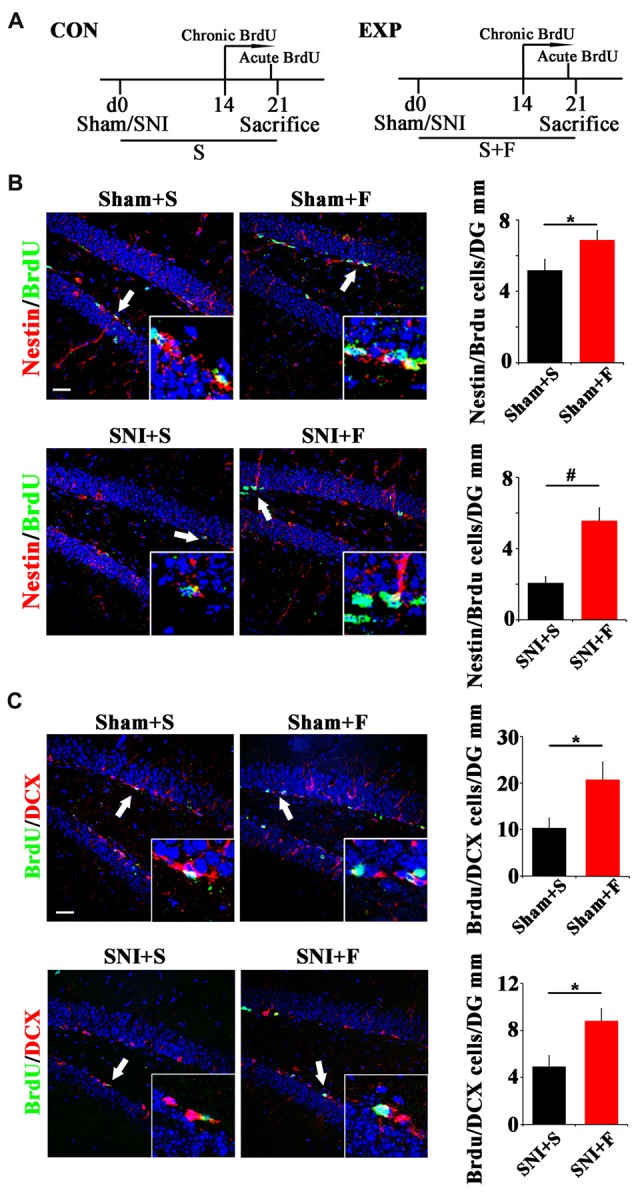
Effects of Fluoxetine treatment on the NSC proliferation and neurogenesis in hippocampus. **(A)** Schematic drawing of the experimental design. **(B)** Double-immunostaining of BrdU/Nestin in sham mice treated with saline (Sham+S), sham mice treated with Fluoxetine (Sham+F), SNI mice treated with saline (SNI+S), and SNI mice treated with Fluoxetine (SNI+F). Notice that Fluoxetine treatment significantly increased the number of BrdU/Nestin-positive cells in both sham and SNI-treated mice after acute BrdU incorporation, as compared to corresponding saline controls. **(C)** Double-immunostaining of BrdU/DCX in sham mice treated with saline (Sham+S), sham mice treated with Fluoxetine (Sham+F), SNI mice treated with saline (SNI+S), and SNI mice treated with Fluoxetine (SNI+F). Notice that Fluoxetine treatment significantly increased the number of BrdU/DCX-positive cells after 7 days’ successive BrdU administration. CON, control group. EXP, experimental group. S, saline. F, Fluoxetine. Inserts in **(B,C)** are typical double-stained cells in each group, which were pointed by arrows. Bars = 50 μm. Values represent mean ± SE. Unpaired, two tailed Student’s *t*-tests were performed in **(B,C)**. *P < 0.05, ^#^*P* < 0.001.

To further explore the involvement of active NSCs in the pain-associated anxiety, we next investigated whether clinical anxiolytic could affect the behavior of NSCs and Wnt signaling in hippocampus. Fluoxetine, the most widely used anxiolytic in clinic, was administered to mice for 3 weeks starting from the day of SNI as described (Miller et al., 2008). Acute or chronic BrdU incorporation was conducted at 21 dpi or from 14 dpi to 21 dpi, respectively (Figure 5A). As compared to saline controls, Fluoxetine treatment significantly increased the number of Nestin/BrdU-positive cells after acute BrdU-incorporation in both sham- and SNI-treated mice (*P* = 0.049, *P* = 0.0004 respectively. *n* = 4 mice per group; Figure 5B). In line with this result, the number of BrdU/DCX-positive cells increased significantly in Fluoxetine-treated mice (*P* = 0.046, *P* = 0.035 respectively, *n* = 4 mice per group; Figure 5C).

**Figure 6:**
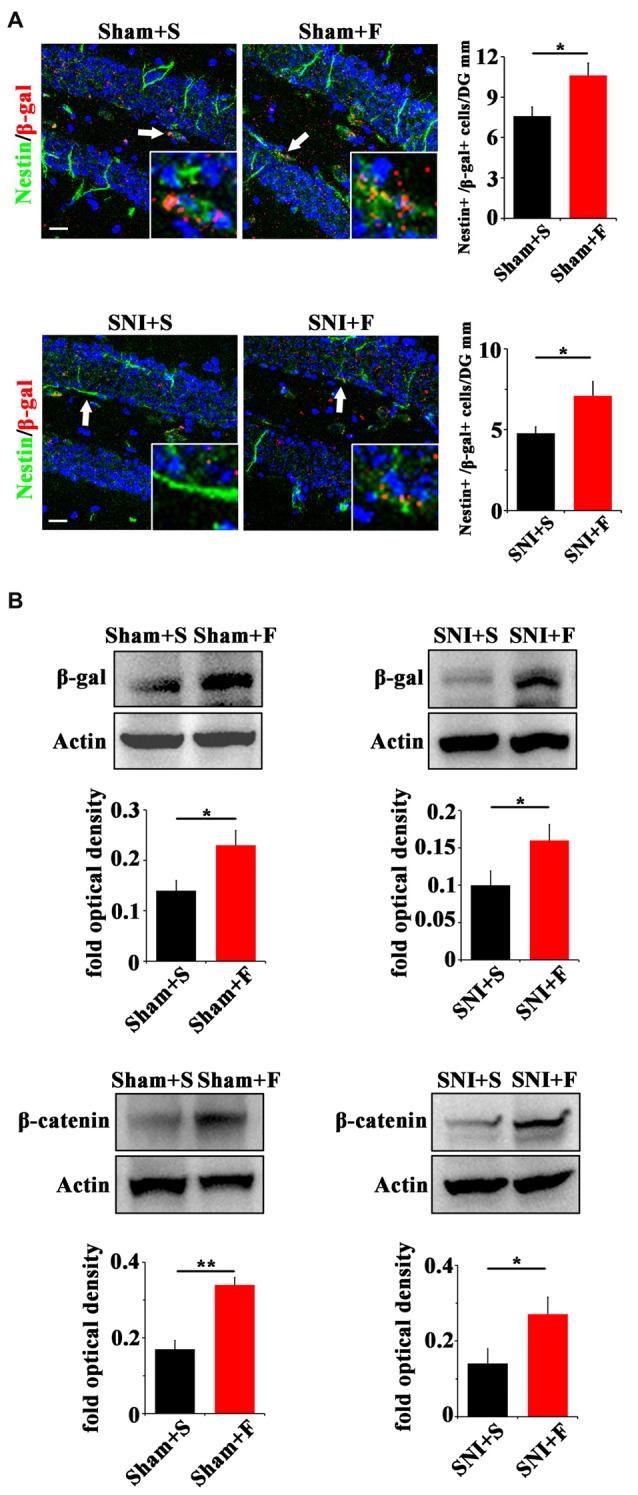
Effects of Fluoxetine treatment on the Wnt activity in hippocampus.** (A)** Double-immunostaining of Nestin/β-gal in sham mice treated with saline (Sham+S), sham mice treated with Fluoxetine (Sham+F), SNI mice treated with saline (SNI+S), and SNI mice treated with Fluoxetine (SNI+F) at 21 dpi. Notice the significant increase of radial Nestin-positive cells in Fluoxetine treated mice. **(B)** Western-blotting of β-gal and β-catenin in Topgal mice with the following treatments: sham injury plus saline (Sham+S), sham injury plus Fluoxetine (Sham+F), SNI plus saline (SNI+S) and SNI plus Fluoxetine (SNI+F). Notice that Fluoxetine treatment significantly increased the expression level of β-gal and β-catenin in both sham and SNI-treated mice, as compared to corresponding saline controls. S, saline. F, Fluoxetine. Inserts in **(A)** are typical double-stained cells in each group, which were pointed by arrows. Bars = 50 μm. Values represent mean ± SE. Unpaired, two tailed Student’s *t*-tests were performed in **(A,B)**. *P < 0.05, **P < 0.01.

In regard of Wnt signaling, we first examined the expression of Wnt reporter gene β-gal in Topgal mice after Fluoxetine treatment. Fluoxetine treatment significantly increased that the number of radial Nestin/β-gal-positive cells in the vDG of both sham- and SNI-treated mice (*P* = 0.027, *P* = 0.04 respectively. *n* = 4 mice per group; Figure 6A). Western-blotting confirmed the up-regulation of β-gal and β-catenin in Fluoxetine treated mice. (*P* = 0.003, *P* = 0.045 respectively. *n* = 3–4 mice per group; Figure 6B).

### Both Wnt Modulation and Fluoxetine Treatment Do Not Affect Pain Thresholds

**Figure 7:**
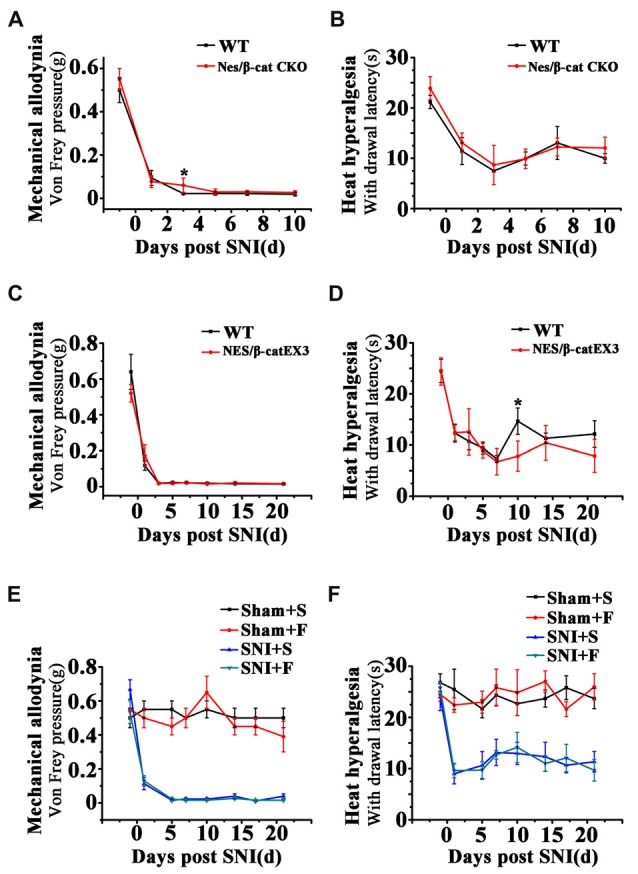
Effects of Wnt modulation and Fluoxetine treatment on pain thresholds. **(A,B)** Effects of ablation of β-catenin in hippocampal neural progenitors on mechanical and thermal withdraw thresholds of SNI treated mice. **(C,D)** Effects of expressing stabilized β-catenin in hippocampal neural progenitors on mechanic and thermal withdraw thresholds of SNI treated mice.** (E,F)** Effects of Fluoxetine treatment on mechanic and thermal withdraw thresholds of sham injured and SNI treated mice. S, saline. F, Fluoxetine. Notice the no change of pain thresholds in mice with β-catenin manipulation or Fluoxetine treatment. Values represent mean ± SE. One-way ANOVA analysis with Dunnett’s *post hoc* test was performed in **(A–F)**. *P < 0.05.

To clarify whether the relief of anxiety by inhibiting active NSCs was related to the change of chronic pain, we next examined the effects of Wnt modulation on chronic pain. The results showed that, in comparison with WT mice, neither Nes/β-cat CKO mice nor Nes/β-catEX3 mice exhibited any significant changes of both the mechanical allodynia and thermal hyperalgesia after SNI (Figures 7A–D). Furthermore, no significant changes of mechanical and thermal pain thresholds were observed in Fluoxetine treated mice either (Figures 7E,F). Therefore, it seems that the anti-anxiety effects of Wnt modulation and Fluoxetine are separated with pain modulation.

## Discussion

In the present study, we have investigated the roles of hippocampal active NSCs in the chronic pain-induced anxiety with focus on Wnt/β-catenin signaling. By behavior analysis and morphological examination in Wnt signaling reporting and conditioned β-catenin “loss/gain-of function” mice, we demonstrated that hippocampal active NSCs, not hippocampal quiescent NSCs or the SVZ NSCs, respond actively to chronic pain. Modulating Wnt/β-catenin signaling in adult neural progenitors specifically affected the development of anxiety without affecting the threshold of pain. Our data further demonstrated an enhancing effect of Fluoxetine treatment on the activity of Wnt/β-catenin signaling and the proliferation of active NSCs in hippocampus.

It is known that stress hormones, such as corticosterone, impair hippocampal neurogenesis (Workman et al., 2015). However, the reports on whether chronic pain induces stress hormones are not consistent. Some researchers reported higher levels of corticosterone in human patients with chronic back pain (Vachon-Presseau et al., 2013), while others not (Muhtz et al., 2013). In mice, persistent increase of corticosterone has been found in inflammatory pain models (Zheng et al., 2017), while an acute increase or no change of corticosterone was reported in neuropathic pain models (Yalcin et al., 2011; Benedetti et al., 2012). To minimize the effects of stress hormones on the results, we adopted neuropathic pain model in the present study. By examining the morphology of Nestin-positive cells and incorporation of BrdU, we demonstrated a rapid decrease of active NSCs which was followed the appearance of anxiety behaviors, in the vDG of mice suffering chronic neuropathic pain. In our SNI model, no changes of SVZ neurogenesis were detected even at 21 dpi when most mice showed anxiety behavior. These data were in consistent with current knowledge that ventral hippocampal but not SVZ was a key brain region involved in mood regulation.

Furthermore, Fluoxetine treatment promoted the proliferation of active hippocampal NSCs, but not the quiescent hippocampal NSCs and SVZ NSCs. These data, in together, suggested that hippocampal active NSCs may be the major population of adult NSCs which may be involved in the pain associated anxiety.

The quiescent and active NSCs in hippocampus are regulated by different niche signals. BMP and Notch signaling have been demonstrated to maintain the long-term cell cycle and the neurogenic potential of quiescent NSCs (Mira et al., 2010; Llorens-Bobadilla et al., 2015). Wnt and Shh signaling act mainly on active NSCs (Han et al., 2008; Wang et al., 2011). Inhibiting hippocampal neurogenesis using BMP and Noggin transgenic mice which regulate BMP signaling resulted in blockage of chronic pain but did not affect pain-induced depression (Apkarian et al., 2016). In our study, we first demonstrated reduction of Wnt activity in chronic pain model by using a Wnt signaling reporter mouse line and examining the expression of key intracellular molecules in canonical Wnt signaling. Considering that there were multiple Wnts and Frizzled receptors and only hippocampal and SVZ neural progenitors in the adult brain express Nestin, we manipulated the expression of β-catenin signaling using Tamoxifen-induced Nestin-Cre. Our data showed that in hippocampus, depleting or over-expressing β-catenin affected active NSCs but not quiescent NSCs, which was consistent with the function of Wnt signaling in maintaining the active status of NSCs. What’s more, both depleting and over-expressing β-catenin affected the development of anxiety after SNI without influencing the perception of pain. These results indicated an anxiety specific role of Wnt activated NSCs in chronic pain. Our data do not exclude the possibility of decrease of quiescent NSCs in the long term, which may account for the pain-modulating effects of hippocampal neurogenesis reported by others (Apkarian et al., 2016). Further investigations on the subgroup of interneurons derived from active NSCs may shed new insight for disease-associated anxiety. The promoting effects of Fluoxetine on the proliferation of hippocampal NSCs and activation of Wnt signaling was consistent with previous report that Fluoxetine regulates neurogenesis *in vitro* through GSK-3β/β-catenin signaling (Hui et al., 2014), and supported our speculation. Since Nestin is expressed in both SVZ and hippocampus, 1 weak point of our study is that our data could not exclude effects of β-catenin knockout or stabilization in SVZ and the effects of membrane-bound β-catenin in olfactory bulb and granular neurons in dentate gyrus (Valenta et al., 2012). Our data showed that chronic pain did not affect the neurogenesis and Wnt activity in SVZ, suggesting that SVZ neurogenesis may not have a close link with pain-associated neurogenesis. Previous study using ^14^C labeling has demonstrated robust neurogenesis in adult human hippocampus (Spalding et al., 2013). A recent study reported a sharp drop of neurogenesis in adult human hippocampus (Sorrells et al., 2018). Our data and the anti-depression effects of lithium (Richardson and Macaluso, 2017), a Wnt/β-catenin signaling agonist, nevertheless, indicated that regulating Wnt signaling may be valuable for specifically treating anxiety which is associated with pain or other diseases in the future.

## Author Contributions

YZ, LZ and MW conducted most of the experiments, collected and analyzed data. JYang and AL contributed to BrdU staining. HY and BG contributed to behavior tests. XL, YS and JYu contributed to genotyping. SW and YW conceived the study, provided financial support, analyzed data and prepared the manuscript.

## Conflict of Interest Statement

The authors declare that the research was conducted in the absence of any commercial or financial relationships that could be construed as a potential conflict of interest.

## Supplementary Material

FIGURE S1 Behavior assays of Sham-injured and Spared nerve injury (SNI) mice. **(A)** The time resting/moving and the average moving speed in open-field assay. **(B)** The time resting/moving and the average moving speed in elevated plus maze. Notice that there is no significant difference of the time resting/moving and the average moving speed between Sham-injured and SNI mice.

FIGURE S2 Efficiency of Nestin-Cre recombination and effects of NSCs ablation on SNI-induced anxiety. **(A)** Experimental design. **(B)** Double-immunostaining and quantification of YFP/Nestin in Nestin-Cre:ROSA-YFP mice. **(C)** Double-immunostaining and quantification of DCX in Nestin-Cre:ROSA-DTA mice. **(D)** Open-field and elevated plus maze assays of SNI treated WT and Nestin-Cre:ROSA-DTA mice. Notice the reduction of DCX-positive cells and development of anxiety in Nestin-Cre:ROSA-DTA mice. **P* < 0.05, ***P* < 0.01, ****P* < 0.001.

FIGURE S3 Expression of Axin2 mRNA by qPCR. Notice the decrease Axin2 mRNA in the hippocampus of SNI mice. **P* < 0.05.

FIGURE S4 Effects of expressing stabilized β-catenin on neurogenesis and anxiety behavior in naïve animals. **(A)** Experimental design, double-staining and quantification of DCX/BrdU in Nestin-Cre:β-cateninEX3loxp+/− mice (Nes/β-catEX3 mice). **(B)** Open-field and elevated plus maze assays of WT and Nes/β-catEX3 mice. Notice the no change of basal anxiety behavior of Nes/β-catEX3 mice. ***P* < 0.01.

FIGURE S5 Effects of depleting β-catenin on neurogenesis and anxiety behavior in naïve animals. **(A)** Experimental design, double-staining and quantification of DCX/BrdU in Nestin-Cre:β-cateninloxp/loxp mice (Nes/β-cat CKO mice). **(B)** Open-field and elevated plus maze assays of WT and Nes/β-cat CKO mice. Notice the no change of basal anxiety behavior of Nes/β-cat CKO mice. ***P* < 0.01.
