# Supplementary figures and images for: Anxiety Specific Response and Contribution of Active Hippocampal Neural Stem Cells to Chronic Pain Through Wnt/β-Catenin Signaling in Mice
Source: Front Mol Neurosci. 2018 Aug 24;11:296. doi: 10.3389/fnmol.2018.00296 (PMC6117500; doi:10.3389/fnmol.2018.00296)

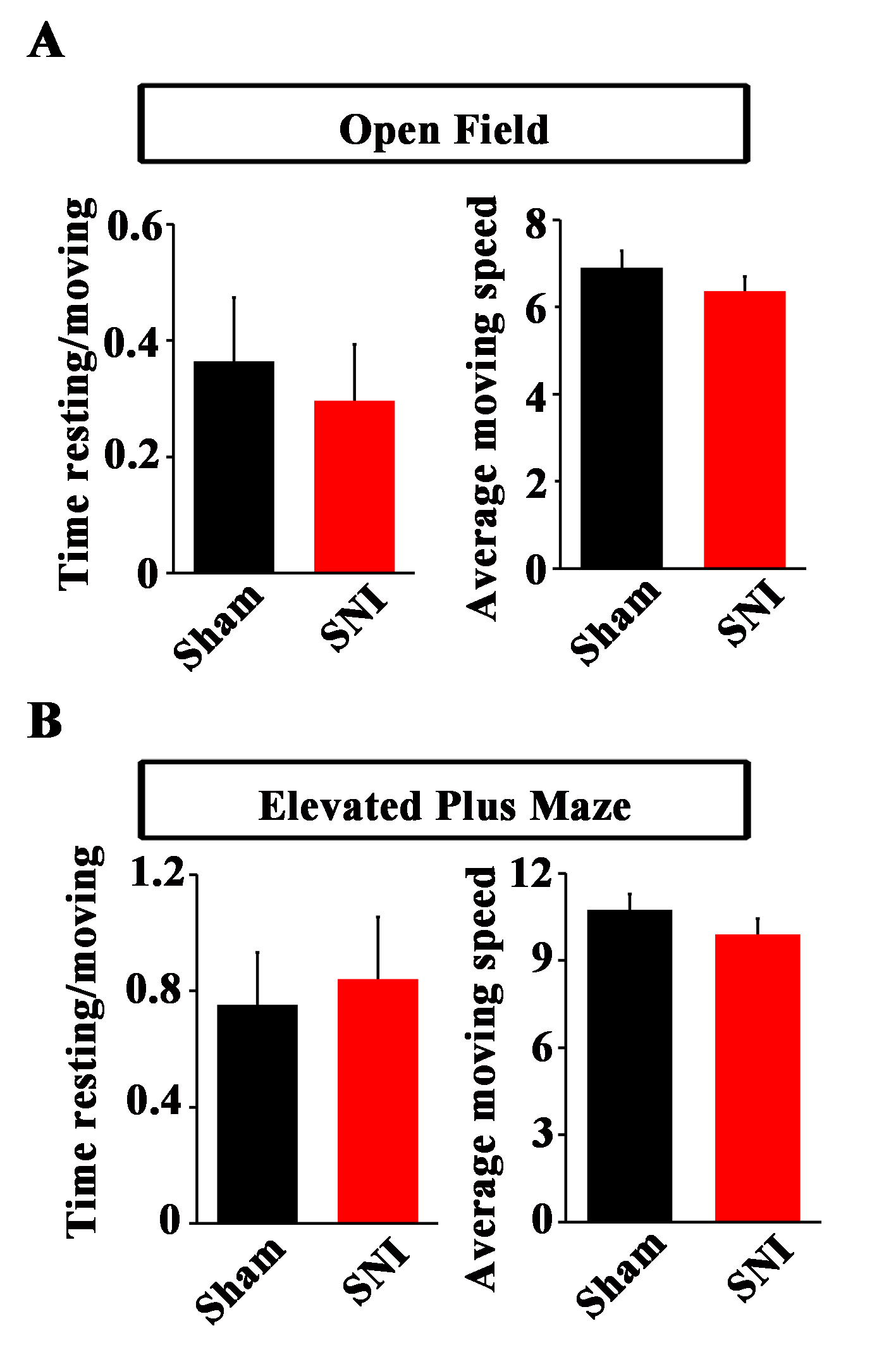

Supplement: FIGURE S1 — Behavior assays of Sham-injured and Spared nerve injury (SNI) mice. (A) The time resting/moving and the average moving speed in open-field assay. (B) The time resting/moving and the average moving speed in elevated plus maze. Notice that there is no significant difference of the time resting/moving and the average moving speed between Sham-injured and SNI mice. [file Image_1.TIF]

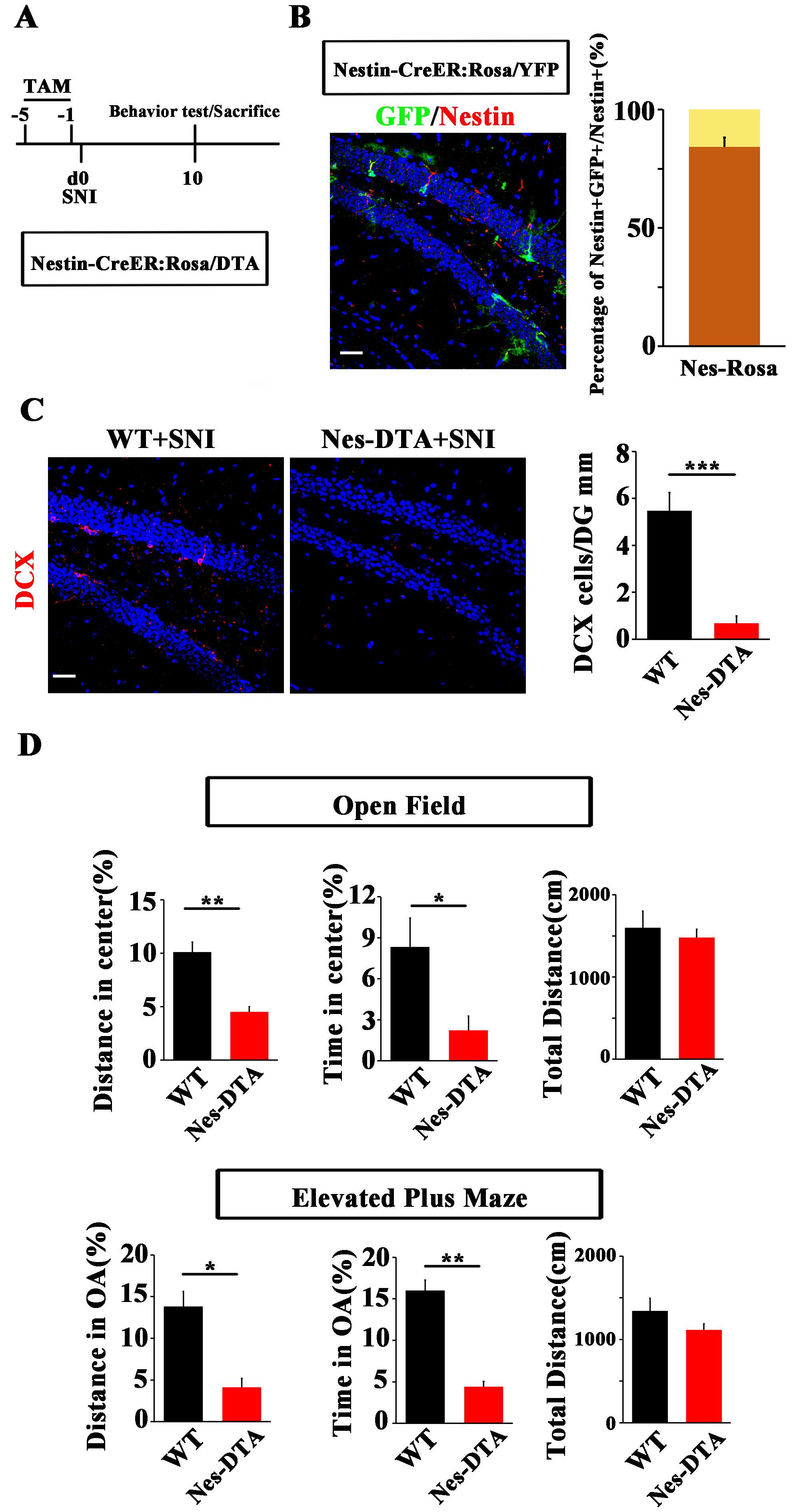

Supplement: FIGURE S2 — Efficiency of Nestin-Cre recombination and effects of NSCs ablation on SNI-induced anxiety. (A) Experimental design. (B) Double-immunostaining and quantification of YFP/Nestin in Nestin-Cre:ROSA-YFP mice. (C) Double-immunostaining and quantification of DCX in Nestin-Cre:ROSA-DTA mice. (D) Open-field and elevated plus maze assays of SNI treated WT and Nestin-Cre:ROSA-DTA mice. Notice the reduction of DCX-positive cells and development of anxiety in Nestin-Cre:ROSA-DTA mice. *P < 0.05, **P < 0.01, ***P < 0.001. [file Image_2.TIF]

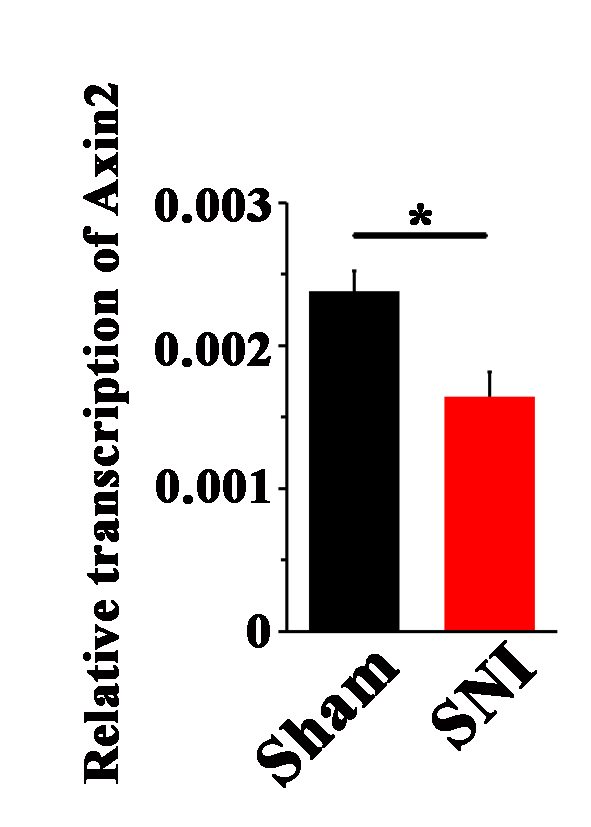

Supplement: FIGURE S3 — Expression of Axin2 mRNA by qPCR. Notice the decrease Axin2 mRNA in the hippocampus of SNI mice. *P < 0.05. [file Image_3.TIF]

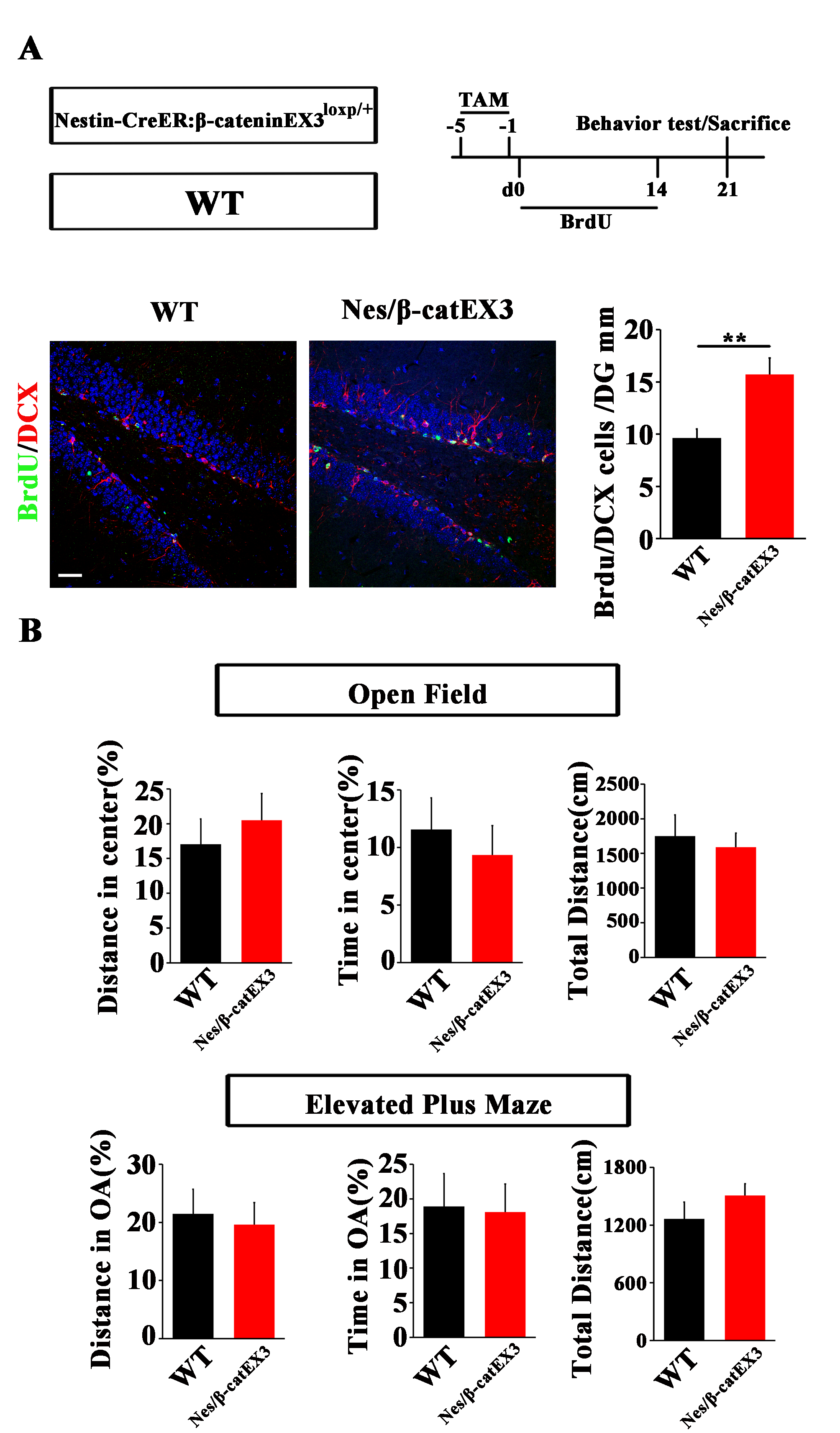

Supplement: FIGURE S4 — Effects of expressing stabilized β-catenin on neurogenesis and anxiety behavior in naïve animals. (A) Experimental design, double-staining and quantification of DCX/BrdU in Nestin-Cre:β-cateninEX3loxp+/− mice (Nes/β-catEX3 mice). (B) Open-field and elevated plus maze assays of WT and Nes/β-catEX3 mice. Notice the no change of basal anxiety behavior of Nes/β-catEX3 mice. **P < 0.01. [file Image_4.TIF]

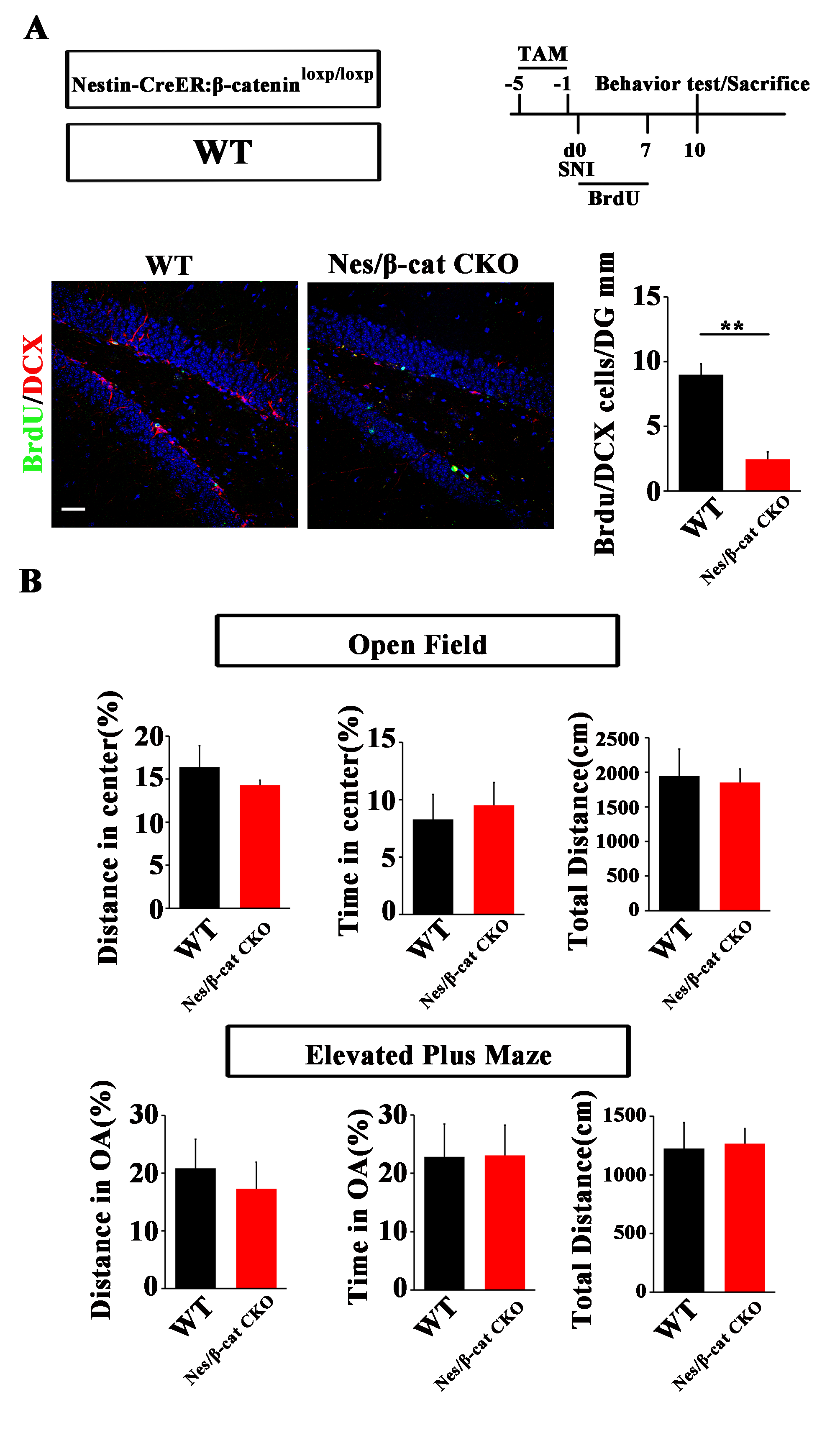

Supplement: FIGURE S5 — Effects of depleting β-catenin on neurogenesis and anxiety behavior in naïve animals. (A) Experimental design, double-staining and quantification of DCX/BrdU in Nestin-Cre:β-cateninloxp/loxp mice (Nes/β-cat CKO mice). (B) Open-field and elevated plus maze assays of WT and Nes/β-cat CKO mice. Notice the no change of basal anxiety behavior of Nes/β-cat CKO mice. **P < 0.01. [file Image_5.TIF]
